# Measuring the Reliability of a Gamified Stroop Task: Quantitative Experiment

**DOI:** 10.2196/50315

**Published:** 2024-04-10

**Authors:** Katelyn Wiley, Phaedra Berger, Maximilian Achim Friehs, Regan Lee Mandryk

**Affiliations:** 1 Department of Computer Science University of Saskatchewan Saskatoon, SK Canada; 2 Faculty of Behavioural, Management and Social Sciences University of Twente Enschede Netherlands; 3 School of Psychology University College Dublin Dublin Ireland; 4 Max-Planck-Institute for Human Cognitive and Brain Sciences Leipzig Germany; 5 Department of Computer Science University of Victoria Victoria, BC Canada

**Keywords:** cognitive assessment, gamification, serious games, Stroop task, reliability

## Abstract

**Background:**

Few gamified cognitive tasks are subjected to rigorous examination of psychometric properties, despite their use in experimental and clinical settings. Even small manipulations to cognitive tasks require extensive research to understand their effects.

**Objective:**

This study aims to investigate how game elements can affect the reliability of scores on a Stroop task. We specifically investigated performance consistency within and across sessions.

**Methods:**

We created 2 versions of the Stroop task, with and without game elements, and then tested each task with participants at 2 time points. The gamified task used points and feedback as game elements. In this paper, we report on the reliability of the gamified Stroop task in terms of internal consistency and test-retest reliability, compared with the control task. We used a permutation approach to evaluate internal consistency. For test-retest reliability, we calculated the Pearson correlation and intraclass correlation coefficients between each time point. We also descriptively compared the reliability of scores on a trial-by-trial basis, considering the different trial types.

**Results:**

At the first time point, the Stroop effect was reduced in the game condition, indicating an increase in performance. Participants in the game condition had faster reaction times (*P*=.005) and lower error rates (*P*=.04) than those in the basic task condition. Furthermore, the game condition led to higher measures of internal consistency at both time points for reaction times and error rates, which indicates a more consistent response pattern. For reaction time in the basic task condition, at time 1, *r*_Spearman-Brown_=0.78, 95% CI 0.64-0.89. At time 2, *r*_Spearman-Brown_=0.64, 95% CI 0.40-0.81. For reaction time, in the game condition, at time 1, *r*_Spearman-Brown_=0.83, 95% CI 0.71-0.91. At time 2, *r*_Spearman-Brown_=0.76, 95% CI 0.60-0.88. Similarly, for error rates in the basic task condition, at time 1, *r*_Spearman-Brown_=0.76, 95% CI 0.62-0.87. At time 2, *r*_Spearman-Brown_=0.74, 95% CI 0.58-0.86. For error rates in the game condition, at time 1, *r*_Spearman-Brown_=0.76, 95% CI 0.62-0.87. At time 2, *r*_Spearman-Brown_=0.74, 95% CI 0.58-0.86. Test-retest reliability analysis revealed a distinctive performance pattern depending on the trial type, which may be reflective of motivational differences between task versions. In short, especially in the incongruent trials where cognitive conflict occurs, performance in the game condition reaches peak consistency after 100 trials, whereas performance consistency drops after 50 trials for the basic version and only catches up to the game after 250 trials.

**Conclusions:**

Even subtle gamification can impact task performance albeit not only in terms of a direct difference in performance between conditions. People playing the game reach peak performance sooner, and their performance is more consistent within and across sessions. We advocate for a closer examination of the impact of game elements on performance.

## Introduction

### Background

In 1886, James Cattell observed that it takes people longer to name the colors and pictures of objects than it does for them to read the corresponding word [1]. This experiment, along with others, paved the way for the development of what Cattell would call *mental tests* and what we now call *cognitive tasks*. On the basis of these and other results, JR Stroop developed a test of cognitive ability in which study participants read the color but not the meaning of a color word aloud [2]. The results revealed an interference effect if the word color and word meaning did not match. Typical cognitive tasks require people to respond to such visual or auditory cues, and data about their responses, often reaction time and accuracy, are collected. These data can then be used to study human cognition, create population norms, and inform medical decisions, such as dementia diagnoses [3].

Cognitive tasks are most useful when collecting high-quality, high-quantity data. However, this is a challenging process. Traditionally, capturing large data sets has been time consuming and expensive, requiring highly trained professionals to administer and score tasks with individual participants. With technological advancements, tasks can now be administered via computers, deployed remotely, and automatically scored [4,5]. This automation makes it easier to collect large quantities of data but raises new concerns about data quality. Many factors influence cognitive test performance beyond cognitive capacity, such as motivation, stereotype threat, and fatigue [6,7]. Cognitive tasks are often repetitive and boring, leading to high attrition rates [8] and suboptimal effort from participants [9,10].

In attempts to improve the quality of data collected by such tasks, researchers have increasingly turned to gamification, with the hope that tasks can be made more engaging through the addition of game elements, such as points and graphics.

### Cognitive Task Gamification

#### Overview

Deterding et al [11] defined gamification as “the use of game design elements in nongame contexts.” In the context of cognitive tasks, this process typically involves layering game elements over an already existing task. For example, the Go No-Go task has commonly been gamified by adding points [12], narrative elements [13], and fun graphics [14] to the basic task.

#### Enjoyment and Motivation

Typically, tasks are gamified with the intent of increasing participant enjoyment and motivation. Nicholson [15] noted that gamification can target both extrinsic and intrinsic motivations depending on the game elements used. Reward-based elements, such as points, achievements, and badges, target extrinsic motivation, whereas elements such as play, exposition, and choice target intrinsic motivation. By targeting motivation, researchers aim to combat attrition and encourage repeated, prolonged play [16-18].

However, there is little examination of whether participants experience increased enjoyment when tasks are gamified. In a systematic review of gamified attention tasks, only 25 of the 74 studies reported results from an evaluation of gameplay [16]. When enjoyment is measured, the research shows mixed results. Some studies have found that gamification increases motivation; for example, participants in a stop signal task study experienced higher enjoyment and more flow-like experiences in the gamified condition (as opposed to the basic task) [19].

Other studies have found that certain game elements, especially thematic or narrative elements, can have a negative effect on self-reported enjoyment of cognitive tasks [8,20,21], possibly due to the “chocolate-covered broccoli” effect [22]. Tasks can only be gamified and retain the important elements of a task. When participants expect a fun game and must still complete a repetitive cognitive task, they may experience even lower enjoyment than if they expected a boring task [20]. Game elements can also be used to introduce other emotions. For example, Levy et al [23] found that some older Jewish participants were uncomfortable with their cooking-themed game as they required making recipes containing pork products.

Do these mixed findings imply that researchers should move away from gamifying tasks? Not necessarily, participants might not *enjoy* assessment games more than a control task, but the data they produced may still be of higher quality.

#### Performance

Groening and Binnewies [24] note that enjoyment is only one way to operationalize motivation, one closely linked to intrinsic motivation. They found that adding achievement-based game elements to a series of simple tasks did not improve self-reported motivation but did improve persistence—when participants could earn achievements, they engaged with a Stroop task for longer before voluntarily switching tasks, compared with when no achievements were available. Similarly, Mekler et al [25] found that when they gamified an image annotation task, participants generated significantly more annotations, despite no reported differences in intrinsic motivation or competence need satisfaction when compared with the basic task.

Adding game elements to a task may improve performance (without affecting enjoyment) in various ways. For example, Jung et al [26] compared the performance of participants who were given a numeric goal (ie, generating 22 ideas) with those who were asked to “do their best.” Participants who were given a specific goal generated higher quantity and higher quality responses. When completing cognitive tasks, participants are often instructed to respond “as quickly and accurately as possible.” This nebulous goal can be clarified and reinforced through game elements that provide immediate feedback such as scoring points for fast reactions or losing points for incorrect responses.

When designing gamified tasks for research and assessment purposes, it may be beneficial to focus on influencing performance rather than on enjoyment. Levy et al [23] noted that changes in emotions can influence cognitive abilities, which may interfere with the collection of valid and reliable data when using games as scientific tools. When Vanden Abeele et al [27] compared 2 games designed to measure psychoacoustic thresholds in preschoolers, they found that the more fully developed and motivating game was able to detect lower thresholds. As another example, Delisle and Braun [28] found that changing a task to resemble a fast-paced videogame normalized the performance of participants with attention-deficit/hyperactivity disorder (ADHD), meaning that participants with and without ADHD performed similarly on a gamified task (but differently on a standard task). In some cases, such an effect may be desired, but it depends on why the task is used and gamified.

### Psychometric Properties of Gamified Tasks

Tasks may also be gamified with the goal of improving the psychometric properties of a task, such as validity (how well a task measures what it claims to measure) and reliability (how consistent the measurement obtained by the task is) [29]. There are also different types of evidence for reliability that must be considered when gamifying cognitive tasks. Internal consistency refers to the stability of the task data within an assessment; for example, the similarity of a participant’s reaction time at the beginning of a task to their reaction time at the end of the task. Test-retest reliability refers to the stability of the task data over time; for example, how similar a participant’s score on a task is at one time point compared with their score on the task a month later.

Typical cognitive tasks are boring, repetitive, and long partly because of the issue of reliability. From one trial to the next, people will perform quite differently, so multiple trials are needed to decrease measurement noise [30]. Adding game elements to a task may change the reliability of its measurement. Participants may be sufficiently engaged that their performance is more stable over time; for example, perhaps only 20 trials are needed for a reliable measure, instead of 200. Friehs et al [19] found that response variability in a gamified stop signal task was lower than that in the nongame version. Shorter tasks would require fewer resources to administer and would reduce the burden on participants, which would be particularly beneficial for clinical and pediatric populations.

Game elements also offer the ability to guide participants’ performance. Most cognitive tasks use measures of reaction time and accuracy, which leads to classic speed-accuracy trade-offs—the faster a participant responds, the less accurate they will be, and vice versa. Individual participants also favor speed or accuracy differently than one another [30]. These behaviors can be manipulated through instructions (eg, asking participants to respond as quickly as possible). Game elements can also indirectly encourage participants to emphasize speed or accuracy, for example, by awarding points or feedback for faster or more accurate responses, generating more consistency across participants [30,31].

### This Study

#### Overview

Few gamified cognitive tasks are subjected to rigorous examination of psychometric properties [16], despite their use in experimental and clinical settings. Parsons et al [32] noted that psychology lacks a standard practice of reporting the reliability of cognitive task measurements. This problem is exacerbated when tasks are adapted, such as gamification. Even small manipulations of cognitive tasks require extensive research to understand their effects [33].

In this study, we sought to research how game elements can affect the reliability of scores on a cognitive task, specifically the Stroop task. As a typical cognitive task that demonstrates robust experimental effects in the general population [34], the Stroop task is well suited for this research.

#### The Stroop Task

Building on the 1886 work by Cattell [1] with cognitive tasks, in 1935, Stroop [2] conducted an experiment in which he asked participants to either name the colors of colored rectangles or name the colors of mismatched words (eg, the word “blue” printed in red ink). Participants responded much more slowly when naming incongruent colored words, a paradigm we now call the Stroop effect [2].

Since Stroop’s first experiment and subsequent development of the experimental protocol [35-37], the Stroop task has become one of the most widely used tasks in both cognitive and clinical psychology [34,38]. Recently, the Stroop task has been gamified for experimental and clinical applications. For example, Groening and Binnewies [39] used the Stroop task to investigate the effects of game elements on participants’ motivation and performance. They found that when points and story elements were added to the task, participants were more persistent (they engaged with the task for longer before switching to a new task) and reported higher motivation. Gomez-Tello et al [40] used gamified tasks as part of a battery of tests for neuropsychological screening of children and found evidence of the Stroop effect in a gamified version of the task. However, previous studies have not considered the reliability of the Stroop effect in a gamified task, either in terms of internal consistency or test-retest reliability. Thus, we have little guidance when gamified tasks can or should not be used in assessments.

We created 2 versions of the Stroop task, with and without game elements, and tested each task with participants at 2 time points. In this paper, we report on the reliability of the gamified Stroop task in terms of internal consistency and test-retest reliability, compared with the control task. We also compared the reliability of these scores on a trial-by-trial basis. Our objective was to demonstrate how game elements can affect the reliability of scores on a Stroop task.

## Methods

### Ethical Considerations

This research project was approved on ethical grounds by the University of Saskatchewan Research Ethics Board (BEH 17-418). The participants were given GBP £6 (USD $8.3 at time of study) compensation at each time point.

### Tasks

The control task was designed using the basic computerized Stroop task described by Macleod [34] and Hedge et al [41] as models. Participants were shown words in the middle of their screen in various colors (red, blue, green, or yellow). The word could be the same as the font color (congruent condition), a noncolor word (lot, ship, cross, or advice; neutral condition), or a nonmatching color word (eg, the word “blue” shown in green; incongruent condition). After each word, participants were asked to press a key corresponding to the font color (z-key for red, x-key for blue, n-key for green, and m-key for yellow). The participants first completed a training exercise to learn each keymap. The task consisted of 240 trials in each condition (congruent, neutral, and incongruent) for a total of 720 trials.

The gamified version was designed to increase reliability by manipulating the speed-accuracy trade-off [30] and improving engagement through game elements. On the basis of prior research, which demonstrated increased enjoyment from points and decreased enjoyment from themes added to a gamified task [20], we focused on adding points-based game elements to the Stroop task. Points-based elements also target extrinsic motivation (rather than intrinsic motivation), which may be more effective in influencing participant performance [24]. We followed the feedback category of the Gameful Design Heuristics from Tondello et al [42], which states that the system should offer users clear and immediate feedback, actionable feedback, and graspable progress.

**Figure 1:**
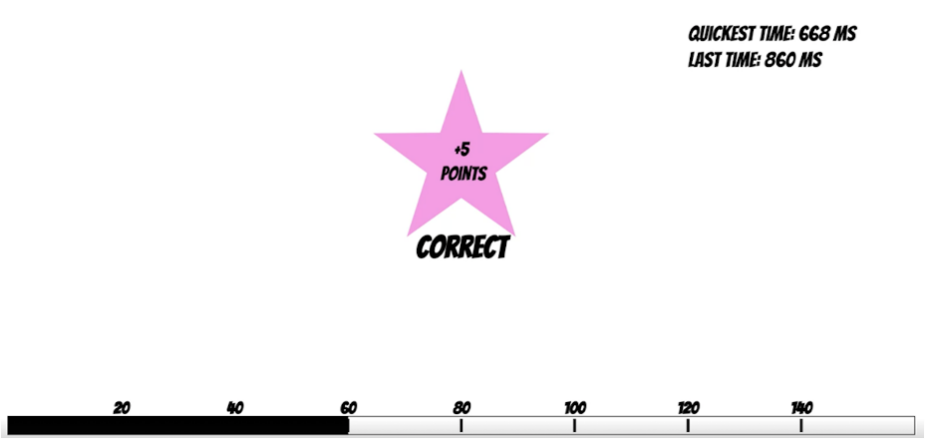
Game version of the task after a correct response was entered.

Using feedback also allowed us to manipulate the speed-accuracy trade-off by preferentially awarding points for faster (but still correct) answers. In the game version of our task, participants saw their response time for each trial and whether they answered correctly. A record of the fastest response time was also displayed at the corner of the screen. They lost 5 points for any incorrect answer, gained 5 points for any correct answer, and were rewarded with a bonus of 25 points for responses that broke their previous “fastest time” record. A progress bar at the bottom of the screen tracked the points (Figure 1).

### Participants

Participants were recruited through Prolific, a web-based platform for recruiting research participants. Web-based platforms are commonly used in human-computer interaction research to conduct studies [43] and have been shown to yield reliable data when precautionary methods for data gathering and analysis are used [44,45]. Each participant completed either the control task or the gamified task at 2 time points, 3 weeks apart (time 1 and time 2). The participants signed a consent form, were given instructions and training for the task, and then completed the task. After completion, they answered questionnaires collecting demographic information, including information about their experience with the task (Intrinsic Motivation Inventory [46]), their general gaming behavior, and self-reported attentional control (Attentional Control Scale [47]).

The study design was between-subjects, with half the participants completing the control version of the task and the other half completing the points version. The participants were randomly assigned to a condition. The study took approximately 40 minutes to complete.

Our analyses were based on the methods of Parsons et al [32] and Hedge et al [41]. Both studies used the same data sets, which had data from 47 (study 1) and 56 (study 2) participants for the Stroop task. In these studies, this sample size was sufficient to observe effects with medium effect sizes. Thus, based on these prior studies, we aimed to obtain approximately 50 participants for each condition [48].

We only analyzed data from participants who had completed both sessions. We also set quality thresholds and removed participants who did not meet them at either time point. Finally, we also removed outlying data points, such as individual trials that were much slower than the average for each participant, to reduce noise in the data, as the study was web-based, and we could not otherwise account for participant distraction from the tasks.

### Statistical Analysis

#### Reaction Time and Error Rate Data

We conducted 2-way ANOVAs with task type (basic or game) and trial condition (congruent, neutral, or incongruent) for reaction time and error rate data. We used 1-way ANOVAs to compare the effect of task type on the skewness and kurtosis of the distribution of reaction time data for each participant. In addition, we conducted 3-way repeated measures ANOVAs (task type × trial type × time) for reaction time cost and error rate cost data. We also created groups representing low and high attentional control based on the median of 51.0 of our participants and then conducted 3-way repeated measures ANOVAs (task type × attention × time) for reaction time cost and error rate cost data.

#### Internal Consistency and Test-Retest Reliability

For measuring and reporting reliability, our analysis followed the recommendations from Parsons et al [32]. To evaluate internal consistency, we used a permutation approach, which involves repeatedly randomly splitting the data, calculating the reliability estimate, and then averaging all estimates. This approach provides a more stable estimate, independent of how trial stimuli and conditions are presented [32]. To evaluate test-retest reliability, we calculated the Pearson correlation between each time point. We also used intraclass correlation coefficients (ICCs) to indicate the degree of consistency and agreement between each time point. On the basis of Parson recommendations, we used ICCs labelled ICC(3,1) and ICC(2,1), as described by Shrout and Fleiss [49]. Finally, we plotted the test-retest reliability as the number of trials increased. To achieve this, we followed the method used by Hedge et al [41].

## Results

### Participants

For the first round of data collection (time 1), we received 135 responses, followed by 78 responses for time 2.

All participants met the criteria for questionnaire speed of completion (participants needed to spend an average of 1.5 seconds per item) and variance (participants needed to show some variance across items). In total, 13 participants were excluded because they too frequently provided an incorrect response on the Stroop task (total incorrect responses>1 SD above the mean number of incorrect responses) and because they responded to trials too slowly (mean reaction time>3 SD above the group mean reaction time). Before calculating the group mean reaction time, we also removed any individual trials that were slower than the average for each participant (reaction time>3 SD above the individual mean reaction time), as well as any remaining outlier trials that were slower than 2000 milliseconds. At time 1, we removed 1667 trials (out of 50,400). At time 2, we removed 1976 trials (out of 49,680). Notably, both at time 1 and time 2, significantly fewer trials needed to be removed from the game condition compared with the basic version; 38.6% of the removed trials were in the game condition at time 1, and 32.9% were in the game condition at time 2.

After exclusions, 65 participants remained (50 female, 13 male, 1 nonbinary, and 1 prefer not to disclose; mean age 23.91, SD 4.64 years), with 31 participants in the basic task condition and 34 participants in the game condition. Our sample had a high proportion of women because of the web-based platform we used [50]. The participants had a mean score of 51.8 (SD 7.54) on the Attentional Control Scale.

### Intrinsic Motivation Inventory

At both time points, the basic task and game conditions showed no significant differences for any of the Intrinsic Motivation Inventory subscales (interest, competence, effort, and pressure).

### Reaction Time and Error Rate Data

We averaged the reaction times and error rates across participants and then analyzed each measure by task type and trial condition at each time point. We also calculated reaction time and error rate costs (mean incongruent trials and mean congruent trials). Table 1 presents the descriptive statistics for each measure.

**Figure 2:**
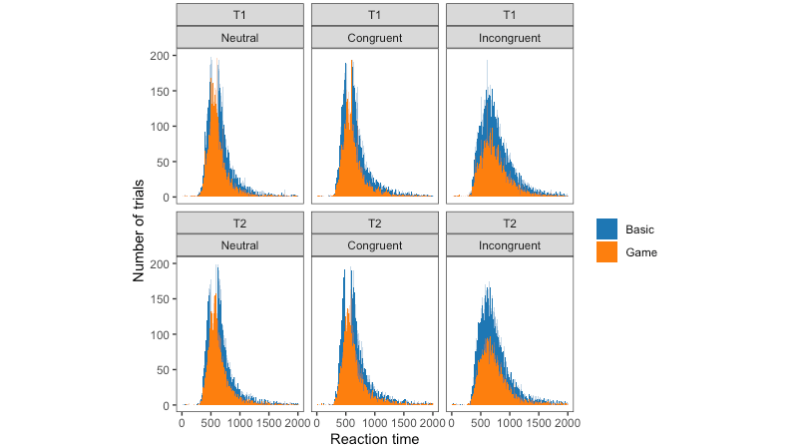
Histograms of reaction time by time point and task type for each type of trial condition.

Histograms of reaction time for all participants are presented in Figure 2 by task type and time point. One-way ANOVAs revealed no significant effects of task type on the skewness and kurtosis of the distribution of reaction time data for each participant (Table 2).

The 2-way ANOVAs for reaction time and error rate demonstrated evidence of the Stroop effect at both time points (significant differences between incongruent trials and both congruent and neutral trials). Furthermore, congruence sequence effect analysis revealed the expected adaptive control effect but no effect of task condition, time, or an interaction between the 2 emerged. There were also significant differences between task conditions at time 1: participants in the game condition had faster reaction times and lower error rates than those in the basic task condition. There were no significant differences at time 2 (Tables 3 and 4).

Two-way repeated measures ANOVAs (task type × time) for reaction time cost and error rate cost data showed no significant interaction effects (Table 5). The 3-way repeated measures ANOVAs (task type × trial condition × time) for reaction time and error rate data showed no significant interaction effects (Table 5). On the basis of grouping our participants into low and high attentional control categories, we found a significant 3-way interaction between time, task type, and attention category for the error rate (Table 5). Participants who scored low in attentional control and were in the basic task condition had a lower error rate cost at time 1 than at time 2. In the game condition, participants who scored low on attentional control had a higher error rate cost at time 1 than at time 2. The error rate cost for participants who scored high on attentional control showed an opposite pattern. There were no significant simple 2-way interactions between task type and attention category at either time point.

**Table 1:** Descriptive statistics for reaction time and error rates, at times 1 and 2 for each task type.

		Time 1, mean (SD)	Time 2, mean (SD)
**Basic task**			
	Congruent reaction time (milliseconds)	678 (103)	659 (104)
	Neutral reaction time (milliseconds)	671 (94.0)	656 (94.7)
	Incongruent reaction time (milliseconds)	796 (124)	758 (118)
	Reaction time cost (milliseconds)	118 (50.9)	98.8 (39.8)
	Congruent correct (%)	96.0 (2.86)	96.1 (2.52)
	Neutral correct (%)	96.7 (2.33)	96.8 (2.43)
	Incongruent correct (%)	93.1 (5.46)	93.6 (4.36)
	Error rate cost (%)	2.86 (4.53)	2.55 (3.23)
**Game task**			
	Congruent reaction time (milliseconds)	638 (94.5)	645 (95.3)
	Neutral reaction time (milliseconds)	628 (84.1)	631 (79.1)
	Incongruent reaction time (milliseconds)	753 (112)	730 (103)
	Reaction time cost (milliseconds)	115 (48.8)	85.3 (42.3)
	Congruent correct (%)	94.6 (3.70)	95.5 (2.50)
	Neutral correct (%)	96.0 (2.53)	96.0 (2.79)
	Incongruent correct (%)	92.1 (3.90)	93.0 (4.80)
	Error rate cost (%)	2.52 (4.71)	2.53 (4.18)

**Table 2:** ANOVA summary table for reaction time distribution.

			Mean squares		*F* test (*df*)		*P* values		Effect size
**Time 1**									
	Skewness	0.316		1.863 (1)		.18		0.029	
	Kurtosis	0.003		0.001 (1)		.98		0.000	
**Time 2**									
	Skewness	0.317		1.852 (1)		.18		0.029	
	Kurtosis	1.159		0.358 (1)		.55		0.006	

**Table 3:** ANOVA summary table for reaction time.

		Mean squares		*F* test (*df*)	*P* value		Effect size
**Time 1**							
	Task type	85,185.015	8.107 (1)		.005	0.041	
	Condition	317,396.780	30.205 (3)		<.001	0.242	
	Task type × condition	49.167	0.005 (2)		.10	0.000	
**Time 2**							
	Task type	23,700.032	2.402 (1)		.12	0.013	
	Condition	201,201.515	20.394 (2)		<.001	0.178	
	Task type × condition	788.555	0.080 (2)		.92	0.001	

**Table 4:** ANOVA summary table for error rate.

		Mean squares		*F* test (*df*)	*P* value		Effect size
**Time 1**							
	Task type	0.005	4.012 (1)		.05	0.021	
	Condition	0.024	18.301 (2)		<.001	0.162	
	Task type × condition	0.000	0.148 (2)		.86	0.002	
**Time 2**							
	Task type	0.002	1.945 (1)		.17	0.010	
	Condition	0.018	15.402 (2)		<.001	0.140	
	Task type × condition	0.010	0.022 (2)		.98	0.000	

**Table 5:** Repeated measures ANOVA summary table for reaction time and error rate.

			Mean squares		*F* test (*df*)		*P* value		Effect size
**Reaction time cost**									
	Task type × time	880.934		1.105 (1)		.30		.017	
**Reaction time**									
	Trial type × task type × time^a^	317.106		0.616 (2)		.49		0.010	
	Attention × task type × time	1325.711		1.665 (1)		.20		0.012	
**Error rate cost**									
	Task type × time	<0.001		0.106 (1)		.75		0.002	
**Error rate**									
	Trial type × task type × time	0.000		0.615 (2)		.54		0.010	
	Attention × task type × time	39.218		5.493 (1)		.02		0.083	

^a^Owing to the interaction violates the assumption of sphericity (*P*<.001), *P* values are derived using the Greenhouse-Geisser statistic.

### Internal Consistency

#### Overview

We estimated the internal consistency of the basic task by using a permutation-based split-half approach [32] with 5000 random splits. Internal consistency ranged between 0 and 1, with higher numbers representing more consistency across an individual’s complete set of trials.

#### Reaction Time

When using the reaction time cost, the (Spearman-Brown corrected) split-half internal consistency for the basic task at time 1 was *r*_Spearman-Brown_=0.78, 95% CI 0.64-0.89. At time 2, *r*_Spearman-Brown_=0.64, 95% CI 0.40-0.81.

For the game condition at time 1, the split-half internal consistency was *r*_Spearman-Brown_=0.83, 95% CI 0.71-0.91. At time 2, *r*_Spearman-Brown_=0.76, 95% CI 0.60-0.88.

**Figure 3:**
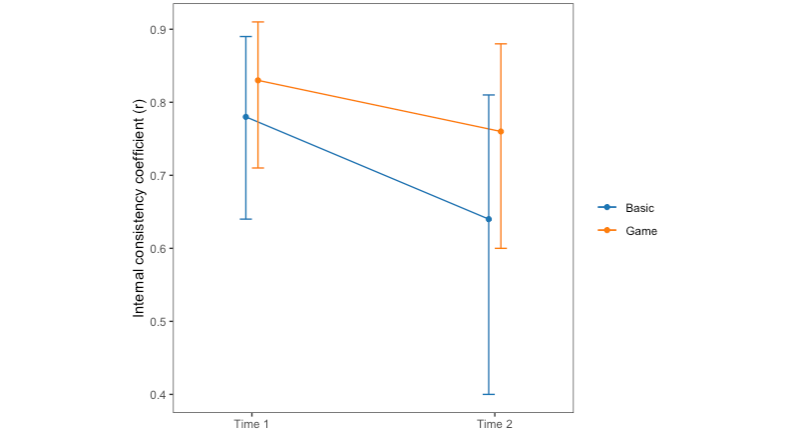
Internal consistency of reaction time cost for each time point and task type.

The internal consistency values were higher at both time 1 and time 2 for the game condition (Figure 3); however, converting the correlations to Fisher *z* scores indicated no significant differences between groups at each time point.

#### Error Rate

When using error rate cost, the (Spearman-Brown corrected) split-half internal consistency for the basic task at time 1 was *r*_Spearman-Brown_=0.79, 95% CI 0.66-0.89. At time 2, *r*_Spearman-Brown_=0.6, 95% CI 0.34-0.79.

For the game condition at time 1, the split-half internal consistency was *r*_Spearman-Brown_=0.76, 95% CI 0.62-0.87. At time 2, *r*_Spearman-Brown_=0.74, 95% CI 0.58,0.86.

**Figure 4:**
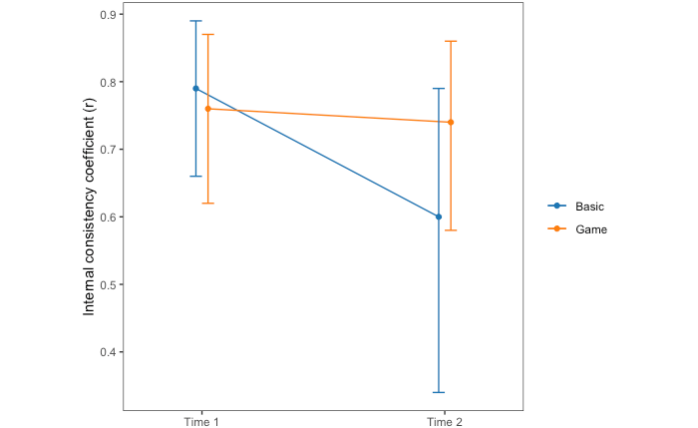
Internal consistency of error rate cost for each time point and task type.

The internal consistency values were higher at time 2 for the game condition at time 2 (Figure 4); however, similar to the reaction time data, converting the correlations to Fisher *z* scores indicated no significant differences between groups at each time point.

### Test-Retest Reliability

#### Reaction Time

Using reaction time cost data, for the basic task, the Pearson correlation between each time point indicated a test-retest reliability of 0.68, 95% CI 0.43-0.84. This correlation was significant (*t*_29_=5.04; *P*<.001). For the game condition, we found a test-retest reliability of 0.58, 95% CI 0.31-0.77. This correlation was also significant (*t*_32_=4.07; *P*<.001).

We also estimated the test-retest reliability between time 1 and time 2 with ICCs using the *psych* package in R (R Foundation for Statistical Computing) [51]. ICCs were used to measure the reliability of a measure between 2 time points. The ICC value can range from 0 to 1, with higher values indicating higher reliability. We report the results of 2-way mixed-effects models for absolute agreement, ICC(2,1), and consistency, ICC(3,1).

Using reaction time cost data, for the basic task, the estimated agreement was 0.61, 95% CI 0.36-0.78, and the estimated consistency was 0.66, 95% CI 0.46-0.80. For the game condition, the estimated agreement was 0.48, 95% CI 0.16-0.69, and the estimated consistency was 0.58, 95% CI 0.35-0.74.

**Figure 5:**
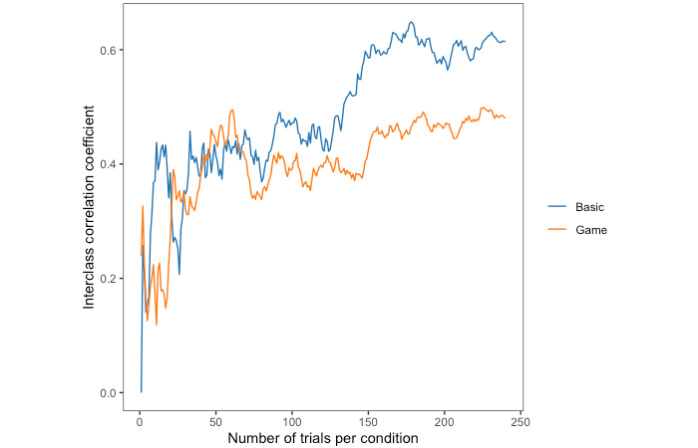
Test-retest reliability of reaction time cost as the number of trials increases for each task type.

Typically, cognitive tasks require many trials to reduce measurement noise. We plotted how ICC(3,1) changes as the number of trials increases, to see if a more stable estimate could be determined with fewer trials when using game elements. Figure 5 shows how the reliability of the Stroop effect (reaction time cost) changes with an increasing number of trials.

**Figure 6:**
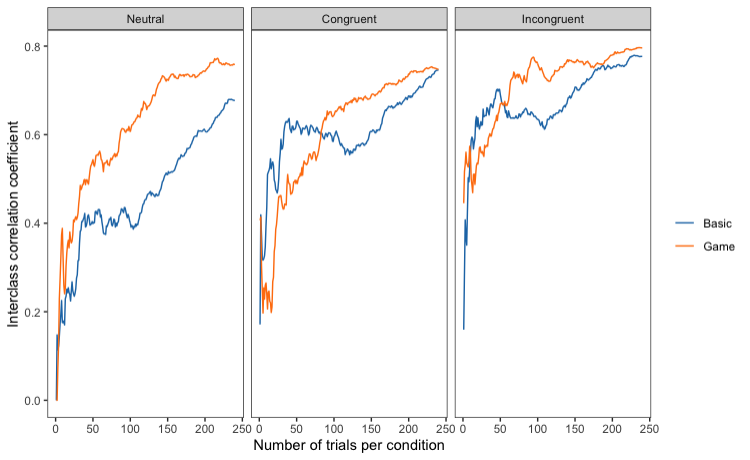
Test-retest reliability of reaction time as the number of trials increases for each trial type and task type.

To investigate why the game condition shows lower test-retest reliability, we also plotted how the reliability of reaction time changes over time for each trial type (neutral, congruent, and incongruent trials; Figure 6). Comparing the plots suggests that the game condition reaches a higher level of consistency sooner for incongruent trials, compared with both neutral and congruent conditions. The basic task showed similar patterns of consistency across all trial types.

#### Error Rate

Using error rate cost data, for the basic task, the Pearson correlation between each time point indicated a test-retest reliability of 0.55, 95% CI 0.24-0.76. This correlation was significant (*t*_29_=3.56; *P*=.001). For the game condition, we found a test-retest reliability of 0.62, 95% CI 0.35-0.79. This correlation was also significant (*t*_32_=4.45; *P*<.001).

Using error rate cost data, for the basic task, ICC(2,1) (estimated agreement) was 0.53, 95% CI 0.28-0.71, and ICC(3,1) (estimated consistency) was 0.53, 95% CI 0.28-0.71. For the game condition, ICC(2,1) was 0.62, 95% CI 0.42-0.77, and ICC(3,1) was 0.62, 95% CI 0.41-0.77.

**Figure 7:**
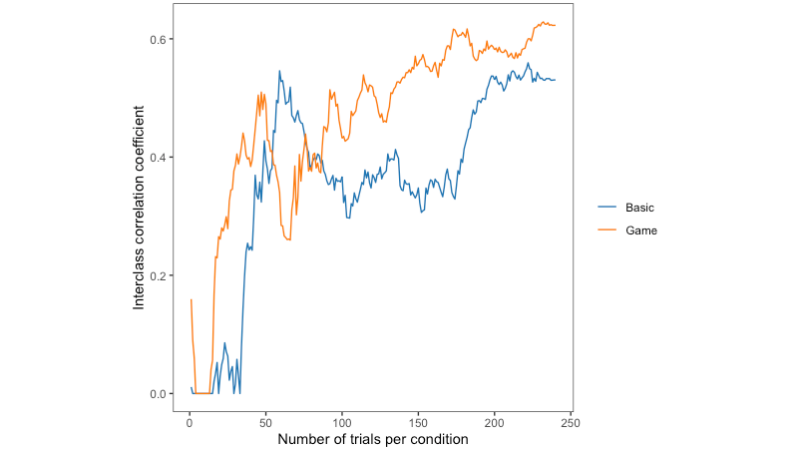
Test-retest reliability of error rate cost as the number of trials increases, for each task type.

We plotted how ICC(3,1) changes as the number of trials increases, to determine whether a more stable estimate could be determined with fewer trials when using game elements. Figure 7 shows how the reliability of the Stroop effect using the error rate cost changes with an increasing number of trials.

**Figure 8:**
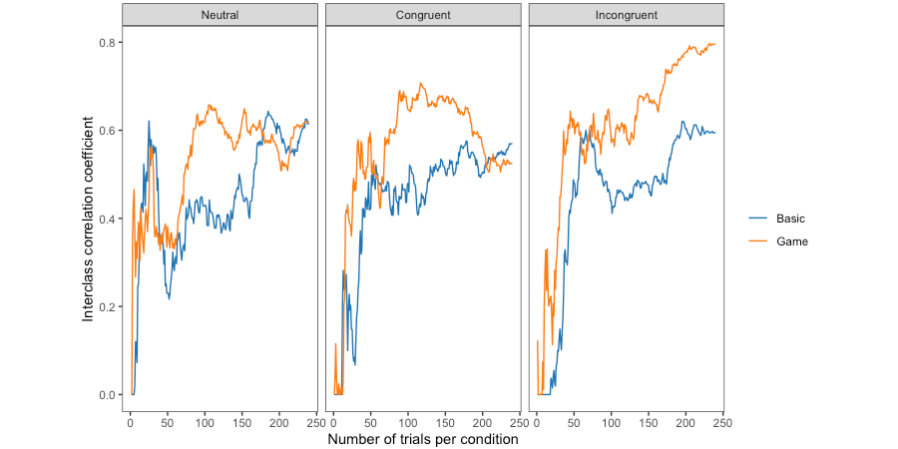
Test-retest reliability of error rate as the number of trials increases for each trial type and task type.

Similar to the reaction time, we plotted how the reliability of the number of errors changes over time for each trial type (neutral, congruent, and incongruent trials; Figure 8). The basic task showed similar patterns of consistency across all the trial types, whereas in the game condition, only the neutral and congruent conditions were similar—the reliability of the incongruent trials continued to increase over time.

## Discussion

### Summary and Explanation of Findings

#### Performance

Both versions of the task demonstrated the Stroop effect, meaning that the effect is robust to the addition of certain game elements. Gamification can affect the validity of cognitive tasks; for example, adding graphics (especially those that change the stimuli participants respond to) can worsen performance compared with a control task [8,12,21]. In this study, in the game condition, reaction times and a progress bar were perpetually displayed on the screen. Graphics indicating gained or lost points also appeared between stimuli. These elements did not interfere with the validity of the Stroop task.

There were no significant differences in performance-based measures between the basic task and game conditions, with one exception: Participants in the game condition had significantly faster reaction times and lower error rates than those in the basic task condition but only at time 1. There may be several reasons for these results.

Points that function as extrinsic motivators have been shown to improve performance in cognitive tasks [25]; however, this effect may be short lived. Nicholson [15] noted that reward-based game elements can drive immediate spikes in engagement but only as long as continuous rewards are provided. In our game condition, participants were continually awarded points for accurate responses; however, for reaction time, they were only awarded bonus points for responses that broke their previous “fastest time” record. There is a physical limitation on how quickly participants can react to stimuli—once that threshold is met, it will be near impossible to improve further, and the motivating influence of the bonus points may be diminished.

In the game condition, participants may also learn faster and reach their “peak performance” sooner. Participants were quickly incentivized to put forth their best effort. This effect may be particularly pronounced when the cognitive demands of the task are higher. When we plotted the reliability of reaction time and error rate as the number of trials increased, the incongruent trials showed an improved pattern of consistency only in the game condition. Specifically, after approximately 50 to 100 trials, the reaction time remained consistent in the game, whereas there was a significant variation in the basic version, with a noticeable drop after 50 trials. A similar pattern was observed for the error rates. For the basic task, the plots of all 3 trial types showed similar patterns across both performance measures. This is especially noteworthy because incongruent trials are arguably the most important trials in the Stroop task, as they are the trials wherein cognitive conflict needs to be resolved. Improved performance in the incongruent trials also explains why the reliability of the Stroop effect (reaction time cost) appeared lower in the game condition—participants in that condition performed better and more consistently in the incongruent trials.

The differences between the basic task and game conditions may be emphasized by incongruent trials because they are more cognitively demanding than the congruent and neutral trials. Evidence suggests that game elements can differentially affect cognition depending on how participants experience the demands of the task. For example, gamification can normalize the performance of participants with ADHD [28].

Another indication of improved performance consistency comes in the form of a significantly smaller number of outlier trials that need to be removed from the game condition compared to the basic version. Approximately twice the number of far-out outlier trials were removed from the basic task. These trials were not considered valuable data and were essentially lost time for both the researcher and the participant. By reducing the number of trials that needed to be removed from performance, the time investment for participants was reduced. Furthermore, this means that the previous results are a conservative estimate of the game’s reliability advantage because the most egregious outliers were already removed from the analysis.

#### Enjoyment

There were also no differences in the self-reported measures of motivation between the basic task and game conditions. These results align with those of other studies, which found that achievement-based game elements are only effective in promoting performance and not motivation [24,25].

Levy et al [23] note how carefully games must be designed to appropriately function as scientific tools and highlight the importance of using the research and data collection goals to inform the choice of game design. For this study, we specifically chose game elements that we thought would influence performance rather than enjoyment. Gamified tasks may be more successful if the game elements are just “good enough” to achieve the goals of the study without interfering with the validity of the task [23]. Because we wanted to improve participant performance irrespective of enjoyment, we did not add extraneous game elements, even if those elements would have made the game more fun.

### Limitations and Future Work

One limitation of our study is the small sample size. The 2 task conditions were designed with subtle differences in the form of points and feedback. While this design was intentional, we also had a relatively small sample size, which may not have been powerful enough to reveal the small effects of our slight manipulation. We recruited 135 participants for time 1 with the intent of having at least 50 participants per condition. However, only 78 participants returned at time 2. It was difficult to incentivize participants to return to a web-based study. Future studies may find significant effects with a larger sample size.

Another limitation is that our sample was heavily skewed toward young adult female participants. We recruited participants through a web-based platform called Prolific. At the time of our study, a young woman made a video describing her hustle as a participant on the platform. Her video went viral on TikTok, resulting in an influx of new signups to Prolific, most of whom were, similar to the creator, female adults in their 20s [50]. However, given the fundamental nature of this research, this sampling bias is unlikely to have influenced the results.

The addition of points and feedback is one simple approach to gamification. Other game elements may produce different results. As discussed, we had theoretical and practical reasons for using points, but even within the category of points and achievement-based game elements, we could have made different design and mechanical choices. For example, adding a leaderboard system may have influenced participant behavior because of increased competition. Mekler et al [25] found that for an image annotation task, participants in the point condition significantly outperformed those in a control condition, where no game elements were used. However, participants in the points condition were, in turn, significantly outperformed by those in conditions where leaderboards and levels were used.

Future studies should investigate other game elements. Other cognitive tasks could also be investigated to determine how game elements affect reliability across task types that target different cognitive domains. Our same methods for investigating reliability could be applied to any gamified task.

### Implications

In this study, we show that the Stroop effect is robust to the addition of simple points-based game elements. Adding points to a Stroop task does initially increase participant reaction time, but this gamification may be most effective in the short term. Our results also suggest that game elements may differently influence *parts* of a cognitive task, such as the more cognitively demanding incongruent trials.

We also provide an example of reporting psychometric data for a gamified task. Despite a long history of cognitive task gamification, the field lacks standard practices regarding how these tasks are made and measured [16]. Any advancement in how these tasks are designed and used requires a stronger base of knowledge on how individual game elements affect cognitive behavioral measures [25,32]. One of the most cited reasons for gamifying tasks is to address the limitations of standard neuropsychological testing [16]; however, these games will never be acceptable replacements for traditional tests if they are not subjected to the same rigorous standards of reliability and validity.

The results of this study suggest a potential advantage of using game-like tasks to assess cognitive functioning, especially for difficult-to-reach populations or individuals who cannot be subjected to prolonged testing. For example, gamified tasks have been shown to provide a more engaging environment that creates a more captivating setting that may aid in collecting data from populations with a lower attention span, such as children or groups of patients with concentration or attention deficits [52].

Our results suggest that the game condition may provide faster onboarding to true performance and improved consistency, as demonstrated descriptively through the lower proportion of outlier trials removed, the reaction time distributions, the split-half internal consistency values for reaction time and error rate, and reaction time cost by trial number charts. This faster onboarding is also supported by the significantly faster reaction times and lower error rates in the game condition at time 1. However, these trends do not result in significant performance differences between the basic task and game conditions in analyses of reaction time cost and also do not influence test-retest reliabilities, suggesting that the game elements we included neither significantly improved nor compromised performance in a gamified Stroop task.

## Abbreviations

ADHD: attention-deficit/hyperactivity disorder
ICC: intraclass correlation coefficient

## Data Availability

The data sets generated and analyzed in this study are available from the corresponding author upon reasonable request.
